# Harnessing the Potential of a Secondary Metabolite-Based Formulation for the Post-Harvest Disease Management and Shelf Life Extension of Banana

**DOI:** 10.3390/metabo16010022

**Published:** 2025-12-25

**Authors:** Karma Beer, T. Damodaran, M. Muthukumar, Prasenjit Debnath, Akath Singh, Maneesh Mishra

**Affiliations:** ICAR-Central Institute for Subtropical Horticulture, Lucknow 226101, U.P., India; kvybhu@gmail.com (K.B.); prasenjitdebnath2@gmail.com (P.D.); akath2005@yahoo.co.in (A.S.); maneeshmishra.cish@gmail.com (M.M.)

**Keywords:** CISH-METWASH, secondary metabolites, shelf life extension, delayed fruit ripening, reduced post-harvest disease

## Abstract

**Background:** Post-harvest losses in bananas, particularly due to diseases such as anthracnose and stem-end rot, significantly limit their storage life and marketability. Developing effective and non-toxic treatments to prolong the shelf life of fruits while maintaining quality is crucial inenabling long-distance transport and facilitating exports. **Methods:** The most popular and commercial banana variety, ‘Grand Naine’, was treated with a proprietary secondary metabolite-based formulation (this refers to a solution containing natural compounds produced by living organisms, which are not directly involved in growth but can influence various biological processes, such as antimicrobial activity) and stored under cold conditions at 13 °C, using vacuum packaging (a method where air is removed from the packaging to reduce spoilage and prolong freshness). Untreated fruits were considered as controls, meaning that they were not subjected to the treatment and served as a baseline for comparison. Shelf life-related parameters such as ethylene production (a plant hormone responsible for triggering fruit ripening), ACC oxidase activity (an enzyme central to ethylene synthesis), respiration rate (the rate at which fruit consumes oxygen and produces carbon dioxide), firmness, total soluble solids (TSS; measures the sugar content in fruit), acidity, and metabolic composition were assessed, including indices of susceptibility to disease. These measurements were taken at regular intervals for both treated and control fruits. **Results:** Secondary metabolite-treated bananas maintained quality for 45 days, staying free from anthracnose and stem-end rot. Control fruits showed over-ripening and an 11.6% percent disease index (PDI). Treated fruits had lower ethylene production (7.80 μg/kg/s vs. 10.03 μg/kg/s in controls), reduced ACC oxidase activity, and a slower respiration rate, delaying ripening. They also had greater firmness (1.45 kg/cm^2^), optimal TSS (13.5 °Brix), balanced acidity (0.58%), and increased flavonoid and antioxidant levels compared to controls. **Conclusions:** Secondary metabolite-based treatment, combined with cold storage and vacuum packaging, extended banana shelf life to 45 days, minimized disease, and preserved fruit quality. This approach substantially reduced post-harvest losses, demonstrating export potential through extended storage.

## 1. Introduction

Bananas are a widely consumed fresh fruit globally, valued for their nutritional properties, including carbohydrates, vitamins, minerals, polyphenols, resistant starch, and antioxidants [1]. Globally, banana is considered the fruit with the highest production, trade, and consumption rates for table purposes as well as processed products. India is the largest producer of bananas, accounting for 25% of global production, with an annual output of 35.36 million metric tonnes [2]. Despite its dominance in production, India ranks 21st in the global banana exports, contributing only 0.3% to international trade (exporting 1.81 lakh tonnes annually), valued at USD 90 million [3]. India, being the largest banana producer globally, faces several challenges in exporting bananas, including logistical constraints, phytosanitary restrictions, and a limited shelf life. As a result, a significant portion of the banana production that does not meet export standards is diverted to domestic consumption, processed into value-added products such as chips, banana powder, or puree, or, in some cases, sold at lower prices in local markets. Unfortunately, a considerable volume is wasted due to inadequate storage and infrastructure for post-harvest processing, accounting for more than 30% of post-harvest losses. Besides these challenges, several restrictions—such as quarantines because of *Fusarium* wilt and viral diseases, different SOPs for post-harvest handling and management requirements as per export standards in different countries, quality production and traceability factors, etc.—limit banana export. To address some of the challenges and extend their shelf life, improve disease resistance, and enhance yields and quality, the present study was undertaken. Banana global exporters include countries such as Ecuador, the Philippines, Costa Rica, Guatemala, and Colombia, which collectively account for 77.96% of the trade [4]. Conversely, on the import side, regions such as the European Union (EU), the USA, China, and Russia account for 73.9% of the total imports [5]. In this context, for India to expand its presence in premium markets like the EU and Russia, it is critical to develop protocols for sea-route exports, ensuring a shelf life of 40–45 days. According to the most recent estimates, its market size is expected to reach USD 147.74 billion by 2030 (https://www.mordorintelligence.com/industry-reports/banana-market), 1 May 2025. The main obstacles hindering its exports include high transportation costs, the early onset of post-harvest diseases, premature ripening during transit, a short shelf life, excessive pesticide residues, and extended transit times [3,6]. At present, most banana exports rely on air transport. However, limited cargo space often creates challenges for exporters. Recognizing this, ICAR-CISH has taken the initiative to develop a protocol for sea-route exports. Exporting by sea typically requires 35–40 days for bananas to reach their destination countries. Therefore, it is essential to enhance the shelf life of bananas to meet the demands of sea-route export. ‘Grand Naine’ is the most widely cultivated banana variety in India. It is a high-yielding Cavendish cultivar, favored for its consistent quality, good post-harvest shelf life, and suitability for both domestic consumption and export markets. Bananas, as climacteric fruits, undergo a ripening process that significantly alters their biochemical and physiological properties, influencing factors such as the appearance, texture, flavor, and aroma [7,8]. Ethylene, a natural plant hormone, plays a pivotal role in regulating ripening, and considerable research has been conducted on blocking its biosynthesis to enhance the shelf life of fruits such as tomatoes, mangoes, apples, and bananas. However, handling and transportation stresses often accelerate the ripening process, leading to issues such as mechanical damage, premature ripening, and post-harvest losses caused by fruit flies, anthracnose, and stem-end rot [6]. These factors reduce the fruit’s quality by the time that it reaches consumers, particularly during export scenarios where the transit duration is longer, such as through the sea route.

Various chemical and non-chemical treatments have been used to delay banana ripening, which include (i) chemical methods involving hydrogen water [9], salicylic acid [10], 1-MCP [11], andmonooxygenase combined with sodium alginate application [12]; CaC2 treatment [13]; gamma radiation [14]; chitosan coating and low temperatures [15] and (ii) physical treatments involving modified atmosphere packaging (MAP) and vacuum packaging, which slow ripening and extend the shelf life during extended transit [16,17]. However, these methods often face challenges related to cost, complexity, and food safety, driving the need for simpler and more accessible solutions.

With respect to post-harvest diseases in fruits, anthracnose (*Colletotrichum musae* in bananas) and stem-end rot (*Lasiodiplodia theobromae* in bananas) are the predominant diseases that cause significant losses (50–80%) [18,19]. *Fusarium musae* affects bananas during post-harvest decay in Berlin only [20]. In the current study, we focused on anthracnose and stem-end rot diseases as the major threats regarding banana post-harvest decay. Traditional management practices to reduce post-harvest diseases, such as alum-based water washes and hot water treatments, combined with bacterial antagonists and biological control agents (BCAs), have demonstrated reasonable effectiveness in different crops [21,22,23]. Furthermore, post-harvest pest management using hot water treatments has been reported to be successful against fruit flies in mango [24]. However, these strategies have limitations related to health and environmental concerns; thus, they do not provide sustainable solutions for minimizing post-harvest losses. Moreover, they are not feasible for banana fruits. As an alternative to the direct use of microbial formulations, antifungal peptide preparations from BCAs/biostimulants have recently been reported for use in *Lactobacillus plantarum* and *Lactococcus lactis*. These have shown promise in combating pathogen loads and reducing ethylene levels, thereby delaying ripening [25,26,27]. However, due to regulatory issues, these cannot be commercialized because they are living organisms.

In the recent past, secondary metabolites from cell-free extracts of BCAs/biostimulants and phytometabolites with antifungal properties have been characterized as key factors that reduce post-harvest pests and disease incidences, as well as enhancing the shelf life. In this context, the ICAR-Central Institute for Subtropical Horticulture, Lucknow, Uttar Pradesh, India has developed a product named CISH-METWASH, an intellectual property (IP)-protected organic wash formulation (copyright: 2614/2024-CO/L) that is composed of organopeptides, lipids, and antifungal and antioxidant molecules. The present study was devised with the aim of evaluating the ability of the CISH-METWASH formulation to delay ripening and reduce disease incidence in cold-stored banana fruit and to characterize the biochemical and metabolic changes induced by the treatment in reducing post-harvest diseases, especially anthracnose and stem-end rot in banana, as well as delaying the ripening process in order to improve the shelf life.

## 2. Materials and Methods

### 2.1. Fruit Materials

A batch of mature green bananas (*Musa acuminata*, AAA group) was procured from banana fruit gardens located in Uttar Pradesh, where good agricultural practices (GAP) were implemented to produce export-quality fruits. The fruits were visually inspected by experts to ensure that they were of the proper size, color, and texture and free from physical damage and fungal infections. Bananas were harvested at 85% maturity, approximately 80–82 days after shooting initiation, to meet export standards. Upon arrival at the laboratory, the hands were immediately sorted and evaluated regarding the initial skin color. Hands that were free of visual defects and of uniform color, size, and shape were selected and cleaned. A sharp knife was used to de-hand the banana fruits.

#### 2.1.1. Harvesting

Harvesting was performed carefully by two people: one cutting the hand and the other placing the bunch on a padded shoulder. A sickle-shaped, sharp knife, free from contamination, was used to create a slant cut. The selected banana fingers were square, closely packed, hard, clean, and free of infestation or mucilaginous threads. They measured 4–5 cm in length and had intact skin without brown spots. Care was taken to avoid latex stains and bruising during handling.

#### 2.1.2. De-Handing

De-handing was performed using a sharp chisel-type de-handing tool, leaving the maximum amount of crown attached to the hand. The crown was cut evenly to prevent the outer finger from detaching. The fruits were subjected to the following treatments before vacuum packing: secondary metabolite-based formulation treatment (TRTMET, 1%) and clean water treatment (TRTCON).

#### 2.1.3. Procedure for CISH-METWASH Treatment (TRTMET)

Banana fruits washed with chlorinated water (1%) were left to dry. Later, they were subjected to a secondary metabolite-based formulation (CISH-METWASH) treatment, which was freshly prepared 10 min prior to use and mixed properly to ensure the uniform distribution of metabolites. CISH-METWASH is a proprietary, organic-based formulation containing bioactive secondary metabolites, such as peptides, phenolic acids, flavonoids, and antifungal lipids (IP-protected in the form of copyright L-158332/2024, dated 18 December 2024). Details regarding the formulation will be available in future publications. The fruits were dipped in a formulation containing secondary metabolites (1%) for 10 min. In contrast, fruits treated with water for 10 min were considered as the controls.

#### 2.1.4. Air Drying

The hands were properly air-dried to ensure that any moisture adhering to the fruit surface and its cut ends was removed. Care was taken to avoid wiping the hands with a cloth or any other material to prevent the removal of metabolites present in the secondary metabolite-based formulation. The formulation was air-dried at ambient temperature (26 °C) to remove moisture from the fruit surface.

#### 2.1.5. Vacuum Packaging, Pre-Cooling, and Storage

Vacuum packaging was performed on individual hands, and they were then air-dried. These hands were packed in 100-gauge polypropylene bags, which were lined in 5-ply CFB boxes holding an average weight of 10 kg. The packed boxes were stacked and loaded into the pre-cooling room, which was maintained at relative humidity (RH) of 85% and a temperature of 13.5 °C, for storage (copyright: L158332/2024). Pre-cooled banana boxes for TRTMET and TRTCON were kept separately in the reefer container, and, for both containers, the storage temperature and relative humidity were maintained at 13.0 °C and 85%, respectively. The protocol, covering the entire process from harvesting to packaging and storage, was developed by the ICAR-Central Institute for Subtropical Horticulture, Uttar Pradesh, India and is protected by copyright L158332/2024.

### 2.2. Assessment of Physical and Biochemical Parameters

#### 2.2.1. TSS Determination

The total soluble solids (TSS) of fruit samples were determined using an ATAGO pocket refractometer (Earma INC., Saitama, Japan). For TSS measurement, a single drop of fruit juice was squeezed from the fruit pulp, filtered through cheesecloth (2 mm pore size), and placed on the surface of a prism in the refractometer. The TSS was then recorded digitally from the instrument in degrees Brix (°B) [28]. Five randomly selected fruit samples from each replication were utilized for TSS estimations.

#### 2.2.2. Determination of Titratable Acidity (TA)

The titratable acidity of fruit samples was determined using the method described in [28]. Five randomly selected fruit samples from each replication were utilized for TA estimations. Approximately 5 g of the fruit sample was homogenized in 50 mL of water using a mortar and pestle and then centrifuged at 8000 rpm for 10 min at 4 °C. After centrifuging (iFUGE-UC02R, Neuation, Accumax Lab Devices Pvt. Ltd., Mehsana, India), 10 mL of the supernatant was collected in a 100 mL conical flask, to which 2–3 drops of phenolphthalein as an indicator were added, and it was titrated against 0.1 N NaOH taken in a burette. The titration endpoint was the appearance of a light pink color, and the value of NaOH in the burette was recorded. Oxalic acid was used as a standard in this experiment, and the acidity was calculated viathe following formula:

$$\mathrm{Titratable}\;\mathrm{acidity}\;\left(\%\right)\;=\frac{\mathrm{Normality}\;\mathrm{of}\;\mathrm{alkali}\;\times \;\mathrm{Equivalent}\;\mathrm{weight}\;\mathrm{of}\;\mathrm{acid}\;\times \;100\;\times \;\mathrm{Titre}\;\mathrm{value}\;\mathrm{of}\;\mathrm{sample}\;\times \;\mathrm{Volume}\;\mathrm{made}\;\mathrm{up}}{\mathrm{Weight}\;\mathrm{of}\;\mathrm{sample}\;\times \;\mathrm{aliquot}\;\mathrm{taken}\;\mathrm{for}\;\mathrm{estimation}\;\times \;1000}$$

#### 2.2.3. Quality Assessment of Fruit Peel Color in Terms of CIELAB Scale

The fruit peel color is one of the parameters that determine the damage and quality of fruit. The peel color of the TRTMET and TRTCON samples was measured using a Hunter Lab device (model ColorFlex, Reston, VA, USA), in reflectance mode (RSIN), and the CIELAB scale (L*, a*, and b*). Five randomly selected fruit samples from each replication were utilized for fruit peel color estimations. The instrument was calibrated using a standard white ceramic tile and a black tile and set up for D65 as the illuminant and with a 10° observer angle. Sampling was carried out by loading the quartz cuvette with the fruit peel sample. The color was determined using the CIELAB color system, where L* indicated luminosity or lightness (L* = 0 for black and L* = 100 for white), and the chromatic parameter a* represented the proportion of redness. On the horizontal axis, a positive a* indicated a red–purple hue; a negative a* indicated bluishgreen. On the vertical axis, b* represented the proportion of yellowness, which varied from blue (−) to yellow (+), as described in [29].

#### 2.2.4. Qualitative Assessment of Post-Harvest Disease Based on Percent Disease Index (PDI)

Anthracnose and stem-end rot are major post-harvest diseases in bananas, caused by *Colletotrichum musae* and *Lasiodiplodia brasiliensis*, respectively [30]. Anthracnose infection on bananas typically begins during fruit development but remains quiescent until the fruit ripens, often manifesting as black, sunken lesions with spore masses or acervuli that are observed during storage and marketing [31]. In contrast, stem-end rot is characterized by a dark, rotting area that develops at the stem end of the fruit, progressing as the fruit ripens. These symptoms were observed and used for PDI estimations. For PDI estimation, five randomly selected fruit samples from each replication were utilized. Screening of the TRTMET and TRTCON samples for disease symptoms of anthracnose and stem-end rot was conducted using percent disease incidence scores. The percent disease index (PDI) was calculated for both anthracnose and stem-end rot diseases using the following formula, as devised in [32]:

$$Percentage\;disease\;index\;(PDI)=\frac{Sum\;of\;all\;numerical\;ratings}{Number\;of\;samples\;observed\times maximum\;disease\;grade}\times 100$$

#### 2.2.5. Estimation of Respiration Rate (CO_2_ Production) and Ethylene Production in Banana Fruits

The respiration rate was determined using an infrared gas analyzer (Fruitron-OMGA-2012) according to the protocol described in [33]. Twelve healthy banana fingers from each replicate were weighed and placed in individual 5 L airtight containers. The change in CO_2_ concentration was determined, and the respiratory rate was expressed as CO_2_ (mg kg^−1^s^−1^) on a fresh weight (FW) basis. Ethylene production from the fruits was determined using a portable infrared ethylene analyzer (Fruitron-OMGA-2012, Navi Mumbai, India). The ethylene production rate was measured and expressed as μg kg^−1^s^−1^ (FW basis).

#### 2.2.6. Sensory Evaluation

The sensory evaluation was performed by 10 panelists consisting of trained and semi-trained staff members who tasted coded samples for three sequential sessions consisting of TRTMET and TRTCON. The qualitatively scored traits such as color, flavor, and overall acceptability using a 9-point hedonic scale consisting of the following: dislike extremely—1, dislike very much—2, dislike moderately—3, dislike slightly—4, neither like nor dislike—5, like slightly—6, like moderately—7, like very much—8, and like extremely—9, as given by [28].

### 2.3. Liquid Chromatography–Mass Spectrometry (LC-MS) Analysis Ofpeel and Pulp of Banana Fruits During Storage

#### 2.3.1. Chemicals

Water (LC-MS grade) and methanol (99.8%, HPLC grade) were obtained from Fisher Chemicals (Fair Lawn, NJ, USA) and NICE Chemicals (Kochi, India), respectively. J.T. Baker (Phillipsburg, NJ, USA) supplied formic acid (90% p.a.) and methanol (99.9%, LC-MS grade). The accurate mass calibration mixture was procured from Thermo Fisher Scientific India Pvt. Ltd. (Mumbai, India).

#### 2.3.2. Sample Preparation

LC-MS analysis was performed on the crude dried extracts of METWASH-treated (TRTMET) and untreated control (TRTCON) pulp and peel samples at different intervals (0, 20, and 40 DOS) in a reefer container at 13 °C and RH 85%. Five randomly selected fruit samples from each replication were utilized. The analysis was conducted at the ICAR-National Research Centre for Grapes, Pune, India. TRTMET and TRTCON banana peel and pulp samples were ground with dry ice to generate a fine powder. These samples were stored at 80 °C in air-tight polypropylene screw-capped tubes until further analysis. Treatment-wise, three biological replicates were prepared for each sample. The banana pulp and peel samples (100 mg each) were extracted with 1.5 mL of acidified methanol/water (7:3, *v*/*v*, 0.1% formic acid). This was performed using an ultrasonic water bath (Spectralab UCB 100, Mumbai, India), which was operated at 40 kHz for 30 min at room temperature. The extract was centrifuged at 10,000 rpm for 5 min. The resulting supernatant was diluted with water (1:1) and vortexed for 30 s. This diluted extract was filtered through a 0.22 μm hydrophilic polytetrafluoroethylene (PTFE) filter and transferred into an auto-sampler vial. The blank sample contained a mixture of water/methanol (1:1, *v*/*v*). Aliquots (100 μL) from each vial were combined to prepare the pooled quality control (QC) sample. Banana sample details: T_1_: TRTCON peel 0 DOS; T_2_: TRTCON pulp 0 DOS; T_3_: TRTMET peel 20 DOS; T_4_: TRTMET pulp 20 DOS; T_5_: TRTMET peel 45 DOS; and T_6_: TRTMET pulp 45 DOS.

#### 2.3.3. Data Acquisition

The sample extracts were injected into a DionexUltiMate 3000 ultra-high-performance (UHP) LC that was coupled with a Q-ExactiveOrbitrap MS (Thermo Fisher Scientific, Mumbai, India) that housed an auto-sampler, a rapid separation column compartment, and a quaternary pump. A reverse-phase (RP) ACQUITY UPLC HSS T3 column (100 mm × 2.1 mm×1.8 μm) (Waters Corp., Bangalore, India) was used to carry out chromatographic separation, where the mobile phase consisted of buffer A, a mixture of acidified (0.1%, *v*/*v* formic acid) water/methanol (90:10, *v*/*v*), and buffer B, a mixture of acidified (0.1%, *v*/*v* formic acid) water/methanol (10:90, *v*/*v*). Thereafter, the samples were acquired in positive heated electro-spray ionization (HESI) mode with a total run time of 25 min. The optimized gradient flow included 1.0 min at 95% (A), followed by a linear decrease from 95% to 5% (A) over 10 min, an isocratic cleaning step for 6 min at 95% B, a linear increase in A to the initial conditions (95% A) over 1 min, and a column equilibration step at 95% A for 7 min. The solvent flow rate was set at 0.4 mLmin^−1^, and the injection volume was 5 μL. Throughout, the column temperature was maintained at 40 °C. The data were acquired in full-scan data-dependent MS/MS (ddMS2) (Top N) mode with no inclusion list. Using a maximum injection time (IT) of 100 microscans (ms), an automatic gain control (AGC) target of 1 × 10^6^, and a resolution power (RP) of 70,000 full width at half maximum (FWHM) (at *m*/*z* 200), the full-scan MS was performed in positive polarity (HESI) between 100 and 1200 *m*/*z*. This was followed by mass fragmentation in ddMS2 mode at a resolution of 17,500 FWHM (at *m*/*z* 200), an AGC target of 1 × 10^5^, and a maximum IT of 50 ms. An isolation window of 4 *m*/*z* (default value) was used with stepped collision energies of 10 (eV), 30 eV, and 70 eV. The chromatograms were processed using the XCalibur™ version 4.1 software (Qual Browser from Thermo Fisher Scientific India Pvt. Ltd., Mumbai, India). Initially, the pooled QC samples were acquired through a series of 10 injections to stabilize the chromatographic and mass-spectrometric outputs. After this, random injections of the biological samples of each variety were acquired, with one pooled injection administered after every six samples. The batch concluded with three additional injections of the pooled QC sample. The predicted metabolites were tabulated, and a comparative analysis was performed between the TRTMET and TRTCON samples of banana peel and pulp.

#### 2.3.4. Data Processing

The raw files were imported into the Compound Discoverer version 3.3 (CD 3.3) software (Thermo Fisher Scientific India Pvt. Ltd., Mumbai, India) for data processing, The untargeted metabolomics workflow was used to align the retention times (Rt), find peaks, predict the elemental composition, and remove background noise (blank subtraction). Noise reduction was performed with the following detection parameters: <5 ppm mass error for both precursor and fragment ions, a minimum intensity of 100,000, an Rt tolerance of ±0.1 min, and considering different adducts, [M+H]+, M^−^, and [M+H+MeOH]+. The filtered features were annotated using mzCloud, Predicted Composition, chemical databases (ChemSpider, Phenol Explorer, FooDB, and PubMed), and a database of flavonoids from the Arita Laboratory of the University of Tokyo, Hongo Bunkyo-ku, Japan.

#### 2.3.5. Chemometric Analysis

SIMCA 14 (Sartorius AG, Göttingen, Germany) was used for statistical analyses, and the processed data from CD 3.3 were row-centered and pareto-scaled. In order to assess the degrees of similarity or dissimilarity among the examined samples, PCA was performed. The most prominent and distinguishing features were identified using pair-wise OPLS-DA models, while the distinguishing metabolites were detected using S-plots. The cutoff values were a *p*-value > 0.05, a log fold change > 2, and VIP values > 1.

#### 2.3.6. Data Analysis, Experimental Design, and Statistical Analysis

The experiments were conducted in a completely randomized design (CRD) based on the factorial concept. Each point of score data was analyzed using the Kruskal–Wallis test at a 0.05 probability level. The Mood median test was performed to delineate the differences between the treatments. Each point of non-scoring data was analyzed by analysis of variance (ANOVA), and mean separation was carried out via Duncan’s multiple range test (DMRT). The standard error was calculated when marking error bars.

## 3. Results

### 3.1. Physical Assessment of Fruit Quality by TRTMET

**Figure 1:**
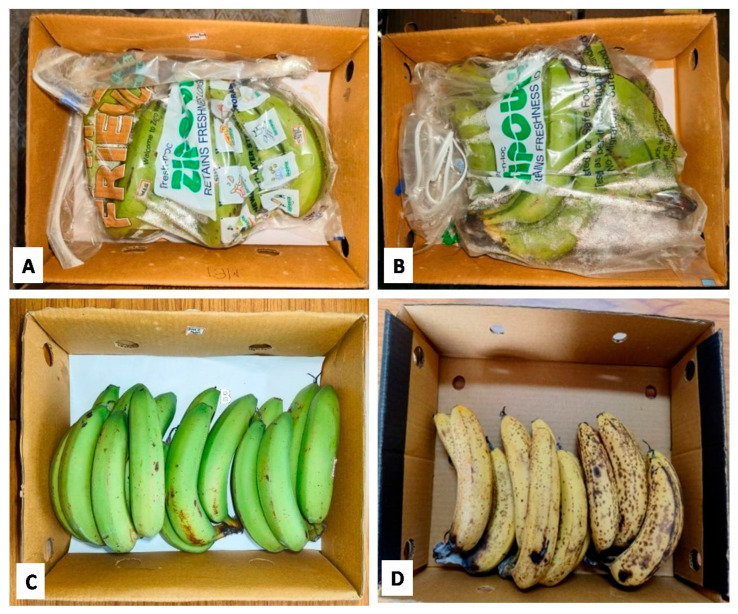
Representative samples of banana fruits withdrawn from reefer container: (**A**,**B**) TRTMET and TRTCON banana fruits after 20 DOS; (**C**,**D**) TRTMET and TRTCON banana fruits after 45 DOS and kept at ambient temperature after 45 DOS for ripening.

During storage in reefer containers, the peel color is an important criterion that reflects the quality and ripeness of banana fruits. Banana fruits without treatment (TRTCON) started color degradation (yellowing) at 20 DOS, while the fruit treated with the secondary metabolite-based formulation retained greenness at 20 DOS. To further quantify the peel color of banana fruits, the L*, a*, b* values were measured, wherein TRTCON fruits recorded values of 73.25, 7.86, and 52.18, respectively, after 20 DOS (Figure 1A–D).

**Figure 2:**
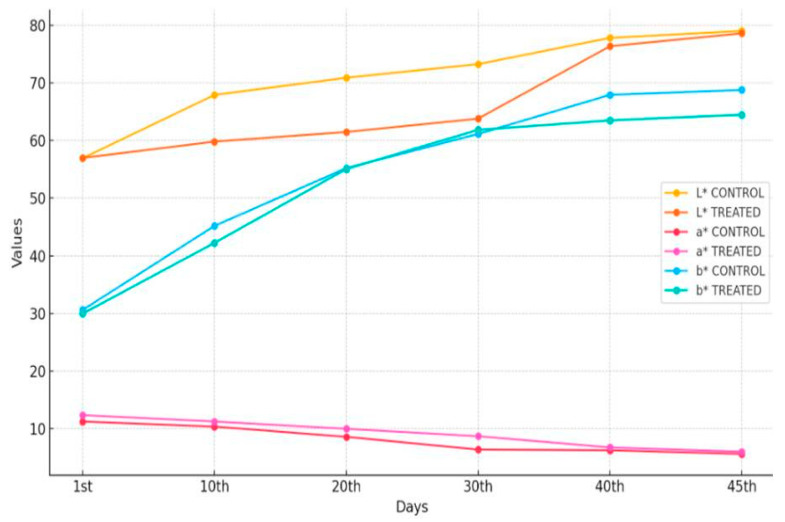
Graphical illustration of changes in L*, a*, b* values in TRTMET and TRTCON banana fruits during storage in reefer containers. Values are mean ± SD of five replications. Values are significant at alpha *p* ≤ 0.05.

Meanwhile, TRTMET fruits recorded L*, a*, b* values of 65.686, 8.914, and 55.212, respectively, which indicated significant changes after 20 DOS in the reefer container. A faster peel color change was noticed in TRTCON banana fruits, but TRTMET fruits showed relatively slower changes in the L*, a*, b values. Avisible difference was found in the withdrawal of fruits after 45 DOS, when the L*, a*, b* values of TRTCON fruits were found to be 76.354, 5.86, and 68.78, respectively, while, in TRTMET, the L*, a*, b* values were significantly different at 79, 5.61, and 67.11, respectively (Figure 2).

**Figure 3:**
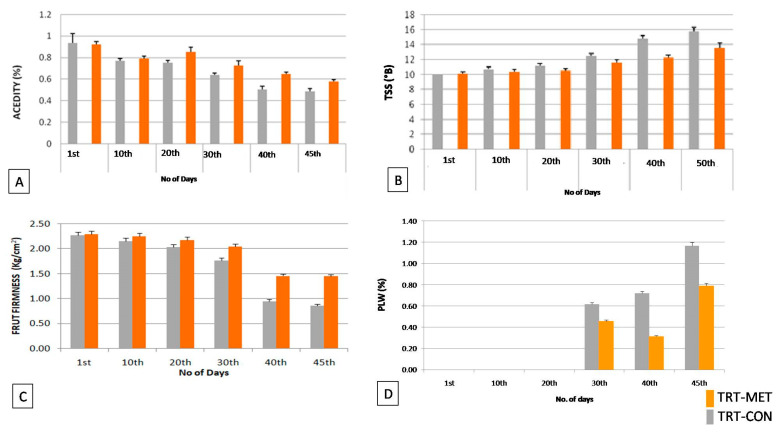
Bar diagram showing the dynamic changes in (**A**) acidity, (**B**) total soluble solids (TSS), (**C**) fruit firmness (FF), and (**D**) physiological weight loss (PLW) of banana fruits. Values are the mean ± SD of five replications. Values are significant at alpha *p* ≤ 0.05.

Generally, low-temperature storage has an impact on fruit firmness (FF) and PLW as the fruits gradually proceed towards ripening. Both parameters play a crucial role in determining the shelf life and export quality of fruits. In the current investigation, it was observed that the FF of TRTMET and TRTCON fruits were 2.38 kg/cm^2^ and 2.27 kg/cm^2^, respectively, at the time of harvesting. After storage in the reefer container, the fruit firmness declined over time. The fruit firmness at 20 DOS was found to be 2.04 kg/cm^2^ in TRTCON and 2.18 kg/cm^2^ in TRTMET. A similar trend was observed at 45 DOS, where both TRTCON and TRTMET showed reduced firmness, at 0.86 kg/cm^2^ and 1.45 kg/cm^2^, respectively (Figure 3C). In the PLW of banana, no significant loss was observed up to a dosage of 20. The PLW was lower in TRTMET fruits (0.78%) compared to TRTCON (1.15%) at 45 DOS (Figure 3D). These results again imply the significant impact of secondary metabolites in enhancing the shelf life by delaying the ripening process with the minimum PLW in banana fruits.

### 3.2. Biochemical Assessment of Fruit Quality Parameters in TRTMET

The acidity levels declined during storage in both TRTCON and TRTMET. Significantly, at 20 DOS, in TRTCON, TA was recorded at 0.752%; in TRTMET, it was 0.854%. However, after 45 DOS, TRTCON and TRTMET samples showed acidity of 0.49% and 0.58%, respectively. TSS was recorded at20 DOS in TRTCON and TRTMET at 11.16 °B and 10.49 °B, respectively. After 45 DOS, TRTCON samples showed TSS of 15.8 °B and TRTMET fruits showed 13.5 °B (Figure 3A). These data revealed that the fruits were gradually moving towards ripening, but the process was significantly slower in TRTMET as compared to TRTCON (Figure 3B).

### 3.3. Reduction in Post-Harvest Disease (PHD) Incidence by TRTMET

**Figure 4:**
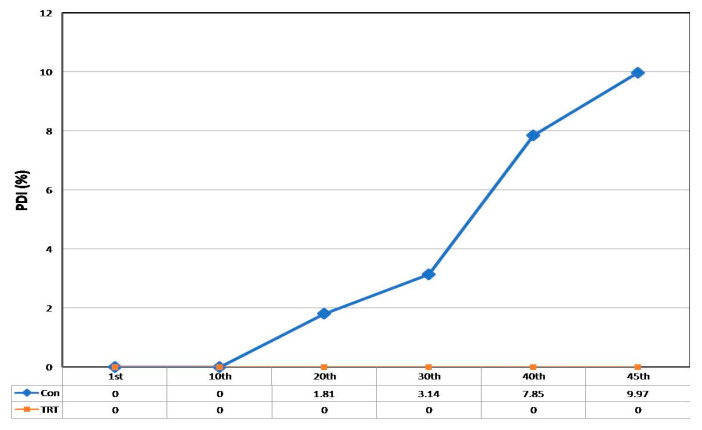
Percent disease index (PDI) inbanana fruits under TRTMET and TRTCON conditions. Values are the mean ± SD of five replications. Values are significant at alpha *p* ≤ 0.05.

At up to 10 days of storage in reefer containers (temperature 13 °C; RH 85%), TRTCON and TRTMET fruits had no symptoms of anthracnose or stem-end rot diseases, but, as the storage period progressed, TRTCON fruits showed symptoms of anthracnose, followed by stem-end rot. At 20 DOS, TRTCON showed disease development (0.81%), progressing by 30 DOS (2.16%) and 40 DOS (5.97%), whereas TRTMET fruits were unaffected (Figure 4). This confirms that the secondary metabolite-based formulation provides protection against pathogens causing diseases.

### 3.4. PCA of Biochemical Parameters Associated with Fruit Quality

**Figure 5:**
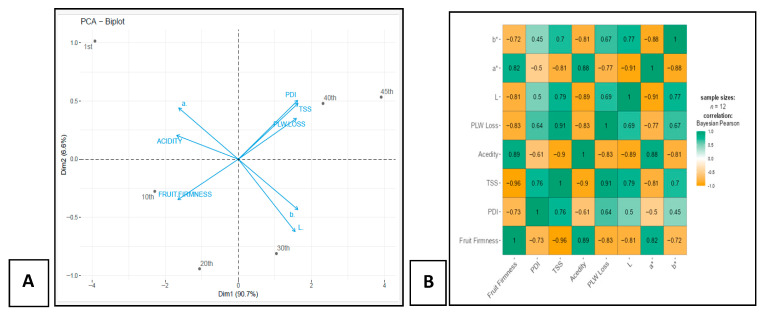
Distribution of physical and biochemical parameters between TRTMET and TRTCON. (**A**) Scatter plot and (**B**) Correlation matrix depicting the relationships among the various parameters (TSS, fruit firmness, PDI, PLW, L*, a*, b*, acidity). Values are the mean ± SD of five replications. Values are significant at alpha *p* ≤ 0.05.

A principal component analysis (PCA) bi-plot represents the relationships between variables and observations in a reduced dimensional space. Here, Dim1 contributes 86.70% of the variance, while Dim2 contributes 7.8% of the total variation. Together, these two components capture the majority of the variance in the data. TSS, PLW loss, and PDI are closely aligned and positively correlated with the component Dim1, whereas fruit firmness and acidity are negatively correlated with Dim1 (Figure 5A). The correlation matrix provides detailed relationships between various fruit quality parameters, such as fruit firmness (FF), PDI, TSS, acidity, PLW loss, and color attributes (L*, a*, b*), in TRTMET and TRTCON fruits. The strongest negative correlation is observed between FF and TSS (−0.96), suggesting that, as FF decreases, the total soluble solids content increases. Similarly, fruit firmness is negatively correlated with acidity (−0.89) and PLW loss (−0.83), indicating that firmer fruits tend to have lower acidity and the minimum weight loss. Furthermore, TSS is positively correlated with PLW loss (0.91) and the color parameter a* (0.87), suggesting that fruits with higher soluble solids content are also more likely to experience greater weight loss and exhibit changes in color. PDI shows a moderate negative correlation with fruit firmness (−0.73), indicating that firmer fruits are less likely to suffer from anthracnose and stem-end rot. Additionally, PDI is positively correlated with TSS (0.76), suggesting that fruits with higher sugar content may be more susceptible to disease development during prolonged storage (Figure 5B).

### 3.5. Comparative Metabolite Profiling of TRTCON and TRTMET Banana Fruits

In order to investigate the differences in the metabolite profiles at different time intervals (0, 20, 45 DOS) in TRTMET and TRTCON banana fruit samples stored in five-ply CFB boxes at regulated conditions in reefer containers, LC-MS based metabolic profiling was performed. The TRTMET banana fruit pulp and peel samples expressed 63 metabolites, which included flavonoids, antioxidants, and defense-related secondary metabolites implicated in delayed ripening and extended shelf life (Supplementary Table S1). Among the metabolites expressed, (R)-2-hydroxyglutarate, 2-C-methyl-D-erythritol4-phosphate, 4-coumarylalcohol, 4-hydroxycinnamoylmethane, 3-amino-3-(4-hydroxyphenyl) propanoate, D-(-)-aspartic acid, indole-3-acetic acid, indole-5, 6-quinone, L-tyrosine methylester, salsolinol, stachydrine, tyramine, and N-caffeoylputrescinewere detected in TRTMET and TRTCON, and these metabolites were classified into the groups of phenols, flavonoids, antioxidants, hormones, and enzymes. However, their expression levels were found to be higher in TRTMET. This experiment was only a qualitative assessment of the metabolite profile.

**Figure 6:**
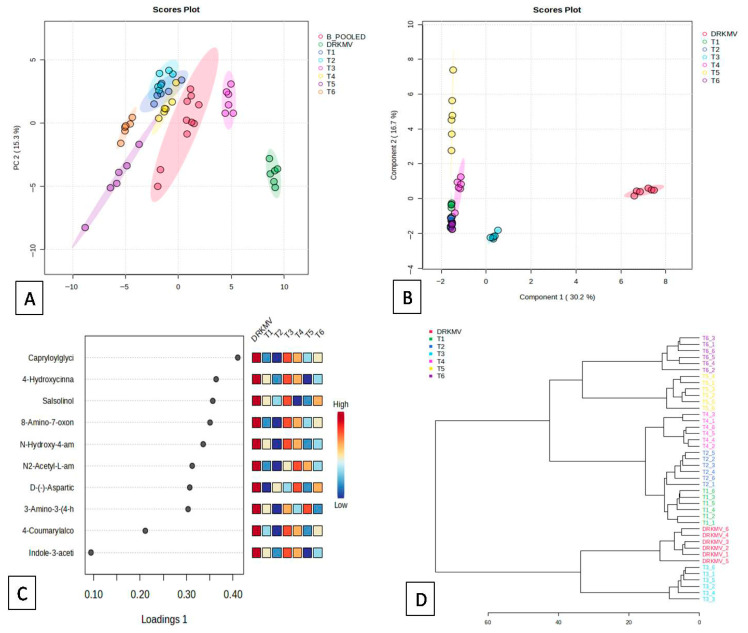
Partial least squares discriminate analysis (PLS-DA) score plot showing moderate clustering of the different treatments along with the control on the basis of the expressed metabolites during the experimental phase at different time points. The color code for the data points of the different treatments is shown in the box. Statistical significance is represented as 95% confidence intervals for each group, with each group comprising six samples. The PLS-DA plots were generated using the top 10 metabolites for each of the components. (T_1_: TRTCON peel 0 DOS; T_2_: TRTCON pulp 0 DOS; T_3_: TRTMET peel 20 DOS; T_4_: TRTMET pulp 20 DOS; T_5_: TRTMET peel 45 DOS; T_6_: TRTMET pulp 45 DOS) (**A**) PCA score plot; (**B**) sPLSDA score plot; (**C**) sPLSDA loading plot; (**D**) Dendrogram.

The PCA scatter plot showed the moderate separation of the expressed metabolites at different time points, where principal components 1 and 2 shared 34.9% and 15.3% of the total variation, respectively. Together, PC1 and PC2 contributed 50.2% of the variation. In the scatter plot, metabolites expressed in the TRTMET peel samples at 20 DOS are represented by violet-colored circles, indicating their distinct placement. In contrast, the metabolites expressed in the TRTMET pulp samples on the same day are positioned differently. Interestingly, by 45 DOS, the TRTMET peel and pulp samples showed clear separation from the metabolites expressed in TRTCON. These results indicate that the secondary metabolite-based formulation (TRTMET) treatment significantly altered the metabolic profile, and these effects were more pronounced over time (Figure 6A).

The partial least squares discriminant analysis (PLS-DA) illustrated the metabolic expression profiles in the TRTMET and TRTCON fruits, in which components 1 and 2 contributed 30.2% and 16.7% of the explained variance. The combined effects of both components 1 and 2 contributed 46.9% of the total variance (Figure 6B). TRTCON (T_1_ and T_2_) at 0 DOS clustered closely, indicating similarity, while TRTMET samples (T_3,_ T_4,_ T_5,_ T_6_) showed distinct clustering, reflecting significant treatment effects that evolved over time from 20 DOS to 45 DOS. The two methods of scattered plotting clearly indicate the unique metabolite expression of the TRTMET samples stored at a low temperature and controlled humidity of 85%.

The PLS-DA loading plots of TRTMET and TRTCON at 0 DOS clearly showed the expression patterns of the top 10 metabolites based on their abundance in the concerned samples at different time periods of investigation. In the TRTCON fruits at 0 DOS (T_1_—peel and T_2_—pulp) of investigation, most of the metabolites were expressed at lower levels. In treatments T_3_—peel and T_4_—pulp for TRTMET at 20 DOS, the maximum metabolites were expressed at higher levels, namely caproylglycin, 4-hydroxycinnamoylmethane, salsolinol, 8-amino-7-oxononanoate, N-hydroxy-4-aminobiphenyl, 4-coumarylalcohol, and indole acetic acid. In contrast, at 45 DOS, TRTMET peel and pulp samples exhibited low to moderate levels of expression (Figure 6C). The dendrogram based on the PCA score plot separated the metabolites into two clusters based on the treatments and days of observation, with the vertical scale (Y axis) indicating a similarity/dissimilarity measure and the individual observations (treatments) on the X axis. T_3_ was clustered with the standards mentioned as DRKMV, which implies that metabolites expressed at 20 DOS in TRTMET fruits were higher in attributes and quite close to the standard (Figure 6D).

**Figure 7:**
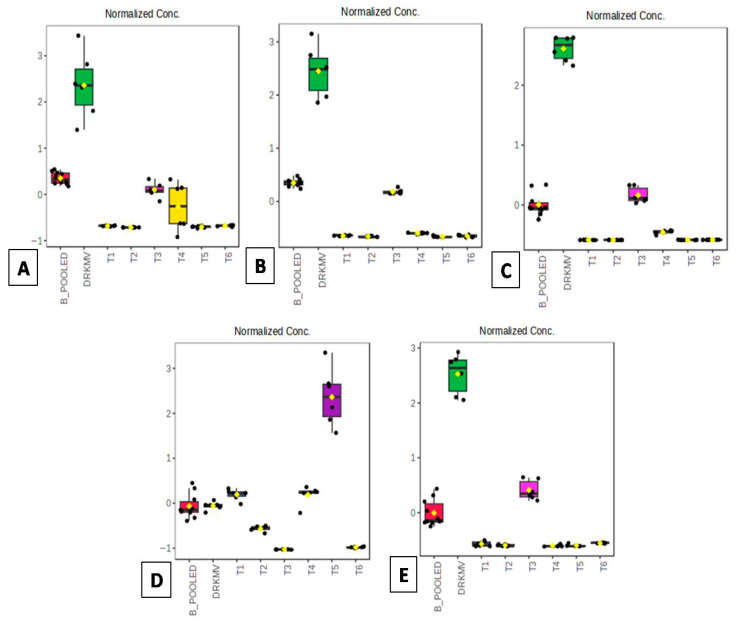
Box whisker plots showing effects of treatment differences among five selected variables. The boxes represent the interquartile range (IQR), and median values are depicted as horizontal lines in the boxes. The top 5 metabolites are shown: (**A**) 4-coumaryl alcohol; (**B**) 4-hydroxycinnamoylmethane; (**C**) caproylglycin; (**D**) tyramine; (**E**) salsolinol. The significantly different metabolites obtained from the ANOVA and post hoc analysis were selected individually, and the related concentrations of each of these were plotted against the six experimental conditions, along with a standard.

A box whisker plot (Figure 7) illustrated important metabolite differences, explained by five selected variables (directly related to fruit metabolism, disease resistance, and fruit ripening). The box plot demonstrated the effects of TRTMET on metabolites’ variability over time for all significantly expressed metabolites (*p* value ≤ 0.05). The experiment identified five metabolites and their expression levels showed distinct variations with respect to different time periods of investigation. 4-Coumaryl alcohol exhibited higher expression levels in treatments T_3_ and T_4_. Conversely, 4-hydroxymethane showed greater variability in peel samples of TRTMET at 20 DOS (T_3_). Significant variability was observed for caproylglycin in treatments T_3_ and T_4_. The tyramine concentration was higher in T_4_ (20 DOS TRTMET pulp) and T_5_ (45 DOS TRTMET peel). Additionally, salsolinol was found at higher levels in peel samples of T_3_ (25 DOS TRTMET peel) but normalized during the later ripening stages in T_5_ and T_6_.

### 3.6. Gas Exchange Relations and Quality Assessment Using Sensory Parameters

**Figure 8:**
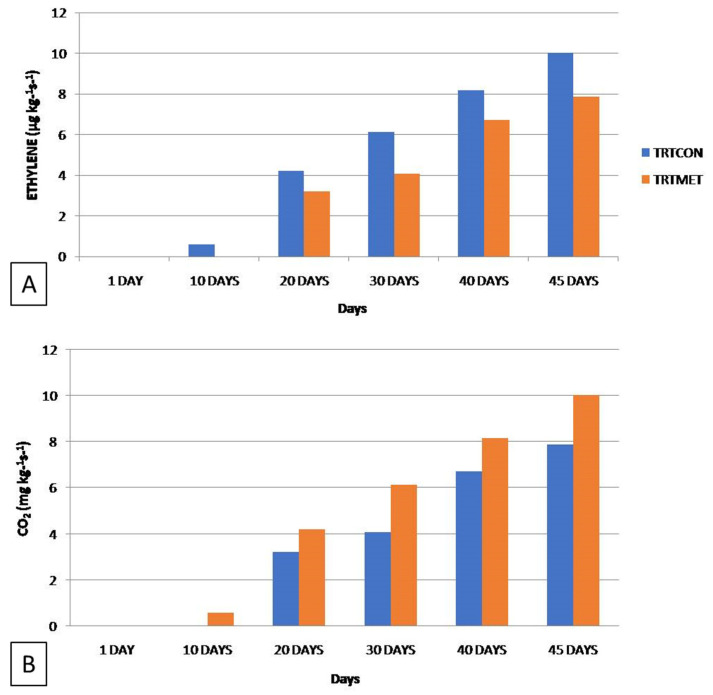
Comparison of gas exchange parameters between TRTMET and TRTCON conditions. (**A**) Fruit respiration (CO_2_) and (**B**) Ethylene (C_2_H_4_) production in banana fruits.

TRTMET fruits showed significantly reduced ethylene production during storage at up to 45 DOS in reefer containers. However, the respiration rate, i.e., CO_2_ release, was increased in the initial days up to 40 DOS, and, after 40 DOS, TRTMET fruits showed a sudden decrease in the respiration rate (Figure 8A,B). The ethylene release rate in TRTCON fruits were observed to be higher than that in TRTMET fruits.

**Figure 9:**
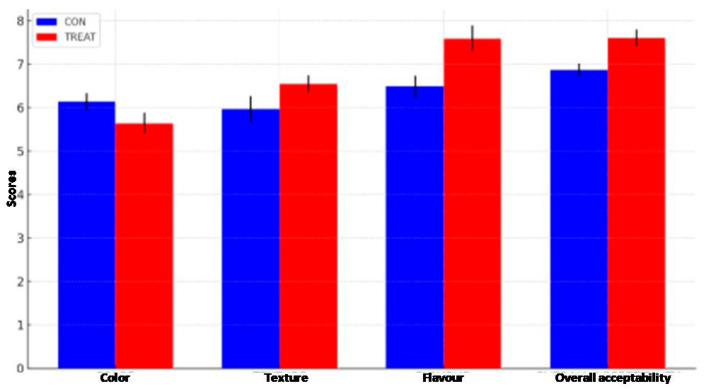
Sensory evaluation of banana fruits withdrawn after 45 DOS. Values are the mean ± SD of five replications. Values are significant at alpha *p* ≤ 0.05.

The sensory quality of banana fruits was evaluated using a nine-point hedonic rating test method [28]. The sensory evaluation was conducted after the withdrawal of TRTMET and TRTCON fruits at 45 DOS, using coded samples for three sequential sessions. The cumulative results showed that TRTMET banana fruits subjected to storage in reefer containers were attractive in terms of texture, flavor, and overall acceptance as compared with TRTCON fruits, as graphically illustrated in Figure 9.

## 4. Discussion

Bananas, being climacteric fruits, continue to ripen after harvest [30] and are controlled by ethylene, which has been demonstrated by using transcriptomics and metabolomics by [34]. It is very difficult to arrest the ripening process in order to enhance the shelf life of bananas. To date, many chemical-based products have demonstrated effectiveness in delaying the ripening process in bananas and other climacteric fruits. Hydrogen water [9], salicylic acid [10], 1-methyl cyclopropane (1-MCP) [11], the exogenous application of melatonin [35], monooxygenase combined with sodium alginate application [12], CaC2 treatment [13], gamma radiation [14], chitosan coatings, and low temperatures [15] have been found to successfully delay ripening. Various post-harvest treatments have also been explored to modulate banana ripening and extend their shelf life. Among them, 1-methylcyclopropane (1-MCP) was found to be the most effective, with 0.5 ppm treatment delaying ripening for over 29 days by inhibiting ethylene action [11]. Hot water treatment using 0.8 ppm hydrogen water also significantly delayed ripening by reducing ethylene production and signaling, allowing storage for up to 26 days [12]. In contrast, calcium carbide, although commonly misused to induce artificial ripening, accelerated the process and led to spoilage as early as the fourth day, posing safety risks and reducing fruit quality [13]. Similarly, gamma radiation at 0.3–0.5 kGy helped to prolong storage to up to 20 days by slowing down ripening [14]. The application of a chitosan coating (1.25%) and low-temperature storage (14 ± 1 °C) demonstrated moderate effectiveness, extending the storage period to 11 and 9 days, respectively [15]. These findings highlight the superiority of ethylene-inhibiting and natural treatments over hazardous ripening agents in extending the post-harvest life of bananas. The treatment examined in the present study surpasses these, as shelf life extension can be achieved for 45 days at 13 °C and 85% RH, while retaining the quality of the fruits.

Moreover, the above strategies are limited in use due to their high costs, complex procedures, food safety concerns, and the effects of chemical treatment. Therefore, the present study presents a formulation that is eco-friendly and cost-effective for direct use in the commercial banana hub, extending the shelf life and reducing post-harvest diseases such as stem-end rot [36], blossom-end rot [20], and anthracnose [37]. However, reports have been published regarding the role of *Fusarium musae* in the post-harvest decay of bananas and its mycotoxin effects on humans [38]. Nevertheless, in the Indian context, *Fusarium musae* is not considered a major post-harvest pathogen for bananas. Moreover, in the current study, fruits were procured from orchards that adhered to good agricultural practices (GAP), where various scientific packages and practices were applied to grow healthy and disease-free crops. Currently, the use of chemicals is a feasible technique for controlling post-harvest diseases; however, it also threatens public health and poses significant environmental risks [39,40]. Therefore, consumer preference for fruits treated non-chemically to remove post-harvest pathogens and using eco-friendly technologies is increasing. *Aloe vera* treatment of banana fruits was reported to significantly enhance the shelf life of bananas by up to four weeks [41]. Meanwhile, chitosan coatings and low-temperature treatment delayed ripening by 11 days and 9 days, respectively [15]. Similarly, plastic bags and banana leaf packaging materials have demonstrated the longest shelf life values of 12 and 8 days, respectively [42]. In a study using cinnamaldehyde (CA) encapsulated in halloysite nanotubes (HNTs), banana fruits stored in PP/HNT-CA nano-composite film bags exhibited the maximum shelf life enhancement of7 days, with significantly lower weight loss, higher firmness, and higher color scores, indicating greater freshness, than bananas stored in neat PP film bags [43]. Secondary metabolites in plants have emerged as a promising foundation for the development of bio-pesticides, offering sustainable alternatives in agriculture. These compounds play vital roles in plant–pathogen interactions. Key secondary metabolites, such as terpenoids, flavanols, and flavones, are stress-inducible phytochemicals that significantly contribute to the development of plant immune responses. When pathogens invade host cells, they replicate and exploit the plant’s biological systems, posing a serious threat to global food security. In response to such stress, plants activate a complex and robust system of growth regulation and defense mechanisms [44]. Plant-derived secondary metabolites and their derivatives have demonstrated significant antimicrobial activity, offering high efficacy with minimal side effects and good tolerance. Unlike traditional antibiotics of microbial origin, these phyto-antimicrobials often operate through distinct mechanisms of action. As a result, they hold strong potential income bating multidrug-resistant microorganisms [45].

The present study has revealed that the TRTMET treatment of banana fruits delays ripening and enhances their shelf life by up to 45 days. This represents the highest-recorded shelf life enhancement, offering scope for long-distance marketing. The TRTMET formulation is a bio-smart organic wash formulation that is rich in secondary metabolites, aimed at delaying early ripening and managing post-harvest diseases (anthracnose and stem-end rot). It is a one-step solution for prolonging the shelf life of bananas, called CISH-METWASH (IP-protected in the form of copyright L-146295/2024, dated 22 January 2024). The process of the application and utilization of CISH-METWASH in the banana standard operating procedure (SOP) is also IP-protected in the form of copyright L-158332/2024, dated 18 December 2024. The process of preparing the formulation and its initial validation were beyond the scope of the present study, as the major focus was on assessing the quality and extending the shelf life using this formulation. The secondary metabolite-based formulation inhibits ethylene biosynthesis by down-regulating ACC deaminase enzyme activity. This formulation is enriched with numerous antifungal and antibacterial metabolites, which play a crucial role in achieving disease-free fruits during prolonged storage at low temperatures. In the present investigation, ‘Grand Naine’ banana fruits were treated with the secondary metabolite-based formulation (TRTMET) and stored at a temperature of 13 °C and RH 90% with vacuum packaging, which helped to increase the shelf life of bananas to up to 45DOS with no disease incidence on the fruit surface in comparison with TRTCON at 20DOS, where necrotic spots developed on the surfaces of the peels, which gradually increased up to 45 DOS (Figure 4). The TRTCON fruits were infected by the fungus early, which triggered rapid ethylene production in the infected fruits, resulting in early ripening and undesirable quality. This phenomenon has been reported previously [46] in green peppers, where the study demonstrated that fungus-infected fruits produced more ethylene. The firmness of TRTCON fruits changed slightly at the early stage of storage and decreased significantly from 20 DOS to 45 DOS (Figure 3C). In contrast, the TSS content of the TRTCON fruits changed slightly at the early ripening stage of storage and then increased gradually from 20 DOS to 45 DOS. The TSS content of TRTMET fruits remained consistently low across all time periods compared to the TRTCON fruits (Figure 3B). During storage, the ripening process continued, resulting in a color change from green to yellow, as well as a faster decrease in firmness and acidity, along with increased ethylene production in TRTCON. In contrast, TRTMET fruits showed delayed ripening (slow respiration and ethylene production rate) through changes in the L*, a*, b* values; the firmness and acidity decreased slowly and there was a gradual increase in TSS. Our results are supported by [9], which showed that hydrogen water delayed the ripening process by reducing ethylene signaling in banana fruits. TRTCON fruits gradually exhibited visual yellowing, while TRTMET fruits remained dark green at up to 30 DOS. After this period, the peel color starts changing from dark green to light green, and this continues up to 45 DOS, at a low temperature (Figure 2). These results are supported by [46], where the authors extended the shelf life to up to 28 days by using differential packing methods and storing fruits at a low temperature of 9 °C.

The quantitative expression of 4-coumaryl alcohol (4CA) was found to be higher in T_3_ and T_4_ TRTMET banana fruits at 20 DOS, while contrasting expression was recorded in T_5_ and T_6_ during the late ripening stage at 45 DOS (Figure 7A). Fruits’ firmness and integrity depend upon the presence of lignin, for which 4CA is the precursor (Supplementary Figure S1). Lignin is a crucial secondary metabolite that contributes to the mechanical strength of horticultural plants and enhances their ability to respond to external environmental changes, including biotic and abiotic stresses. However, the excessive accumulation of lignin can lead to the lignification of horticultural products, reducing their taste quality and nutritional value [47], which is also evident in alfalfa [48]. Surprisingly, it was evident that, at 45 DOS, the expression of 4CA was reduced and returned to an optimal level in both the peel and pulp of banana fruits, confirming the reduction in4CA at the late ripening stage and restricting the lignification process in TRTMET fruits, which progressed to normal senescence afterwards. The 4CA spike at 20 DOS might have activated the lignification process to a greater extent; in contrast, during this period, normal senescence started in TRTCON fruits at 20 DOS onwards. During pathogen infection, callose formation, which aids lignin deposition in the secondary cell walls of fruits, acts as a physical barrier to limit the spread of pathogens [49,50]. This higher lignin formation may have occurred in the TRTMET group, as the pathogen was unable to grow on the fruit surface. Lignin deposition around pathogen penetration sites was found to increase potato’s resistance to *P. infestans* [51]. In tobacco, with lower lignin content, the plant was shown to be susceptible to blackleg and bacterial wilt diseases [52].

Tyramine, a naturally occurring amino acid, was found to be elevated in the late ripening stage in T_5_ at 45 DOS (TRTMET in the peel samples). Tyramine-derived hydroxyl cinnamic acid amides (HCAAs) are a diverse class of secondary metabolites with presumptive roles in plant growth, development, and senescence [53,54,55]. The accumulation of HCAAs has been reported previously as a response to abiotic/biotic stress, wounding, and fungal or pathogen attacks in several plant species [53]. The secondary metabolite-based formulation might have led to an increase in tyramine levels, which in turn enhanced the production of tyramine-derived hydroxycinnamic acid amides (HCAAs) on the fruit peel surface. This increase could be attributed to the creation of an unfavorable environment for pathogen growth on the fruit surface. This enabled fruits to be free from disease at 45 DOS (Figure S1).

Naturally, bananas contain large quantities of dopamine and norepinephrine, and, during the later stages of the ripening process, the concentration of salsolinol (SAL) increases [56]. Salsolinol is known to be involved in the black appearance of overripe bananas [56]. The route involved probably entails the oxidation of SAL to a quinone, which couples with other brown pigments to form melanins [56]. The salsolinol content in the TRTMET fruits may have been lower in the late ripening stage at 45 DOS. This could be the reason for the preserved freshness of the fruits treated with the secondary metabolite- based formulation (Figure 7).

Additionally, 4-hydroxycinnamoylmethane (4HCM), a derivative of cinnamic acid, may also contribute to post-harvest quality by serving as an antioxidant and antimicrobial agent [57]. The antioxidant properties, aiding in reducing oxidative stress in stored fruits and vegetables, prolong freshness and delay spoilage. Due to its antimicrobial activity, it may inhibit the growth of pathogens, thereby extending the shelf life of the product. This compound also contributed to enhancing the resistance of plant tissues to degradation during storage. At 20 DOS, 4-hydroxycinnamoylmethane was found to be higher in the peels of TRTMET fruits and reduced in the later ripening stages. The sudden elevation in 4-hydroxycinnamoylmethane led to ROS scavenging activity in the samples, counteracting the oxidative stress generated during fruit storage through its antioxidant properties and hindering the senescence process for a certain period, which ensured a better shelf life and quality. However, in TRTCON fruits, this process may not have occurred, resulting in quality deterioration.

The LC-MS-based metabolic profiling of the TRTMET banana peel and pulp samples identified 63 metabolites, including flavonoids, antioxidants, and defense-related secondary metabolites. These metabolites may be involved in delaying senescence and/or extending the shelf life. A fruit’s natural resistance depends mainly on flavonoids and anthocyanins due to the antioxidant and antifungal properties contributed by their biosynthesis from the phenylpropanoid pathway (PPP) [58]. Aspartyl-phenylalanine could induce the fruit’s natural defense response and tolerance to fungal pathogens. The post-harvest application of phenylalanine (a precursor of PPP) to mango and avocado fruit reduced anthracnose and stem-end rot caused by *Colletotrichum gloeosporioides* and *Lasiodiplodia theobromae* [58]. Similar results were observed in the present study using banana fruits with TRTMET. Thus, the induction of the defense response infruits is triggered by plant activator compounds that act on the fruit by activating defense mechanisms and increasing their tolerance to fungal pathogens [59,60]. Stachydrine was detected in TRTMET fruit samples; this may influence the synthesis of important secondary metabolites, such as flavonoids, crucial for plant defense. Stachydrine has been reported to regulate plant growth, promoting development by modulating both primary and secondary metabolic pathways, including resistance to specific pests [61,62]. Low-molecular-weight metabolites, such as 2-C-methyl-D-erythriol-2,4-cyclodiphosphate (MEcPP), have recently been proposed to function as signaling molecules in plant responses to environmental stresses [63]. The LC-MS-based metabolomics approach has demonstrated the potential of secondary metabolite-based treatment, combined with low-temperature storage and vacuum packaging, in delaying ripening and enhancing the shelf life of banana fruits. The secondary metabolite-based treatment significantly delayed the ripening process of the fruit. The treated fruits were less ripe than the untreated control fruits. The elevation of 4-coumaryl alcohol (C_9_H_10_O_2_), 4-hydroxycinnamoyl methane (C_19_H_16_O_4_), caprylolglycine (C_10_H_19_O_3_), and tyramines (C_8_H_11_NO) and reduction sinsalsolinol (C_10_H_13_NO_2_) enable an increase in lignin formation, which will help to maintain the firmness of the fruit. It also contributes to the activation of antioxidant production pathways to scavenge ROS, obstruct post-harvest senescence, and restrict disease development on the fruit surface. Furthermore, organoleptic tests confirmed that the TRTMET fruits remained brighter and spotless and retained an appealing taste during long-term storage. This study presents remarkable progress in extending the shelf life of bananas by more than 45 days using a secondary metabolite-based formulation, combined with low-temperature storage (13 °C) and vacuum packaging in CFB boxes within a reefer container.

## 5. Conclusions

The current study achieved the highest shelf life extension and reduced post-harvest diseases in banana fruits. The described secondary metabolite-based formulation is also the first global report to demonstrate the shelf life extension of the commercially viable banana variety ‘Grand Naine’ by more than 45 days, allowing a large quantity of banana fruits to be exported from one country to another. From the present investigation, it can be understood that novel and innovative approaches such as bio-smart technologies will serve as a crucial strategy in combating post-harvest diseases and increasing the potential of the export market for Indian fruits. The use of this formulation, along with controlled temperatures and RH with vacuum packaging, enhanced the shelf life of banana fruits. This non-chemical-based technological invention could facilitate the sea-route shipment of bananas to the international market in larger volumes. Sea-route shipments have longer transport durations, necessitating an extended shelf life so that the fruit can remain fresh for a longer period, retaining its quality.

## Data Availability

All relevant data generated in the study have been reported in the manuscript, and the raw data are available from the authors.

## Supplementary Materials

The following supporting information can be downloaded at: https://www.mdpi.com/article/10.3390/metabo16010022/s1. Table S1: Compounds and Figure S1: Pathways.
