# Supplementary figures and images for: Harnessing the Potential of a Secondary Metabolite-Based Formulation for the Post-Harvest Disease Management and Shelf Life Extension of Banana
Source: Metabolites. 2025 Dec 25;16(1):22. doi: 10.3390/metabo16010022 (PMC12843891; doi:10.3390/metabo16010022)

**A**

# Phenylalanine, Tyrosine and tryptophan biosynthesis

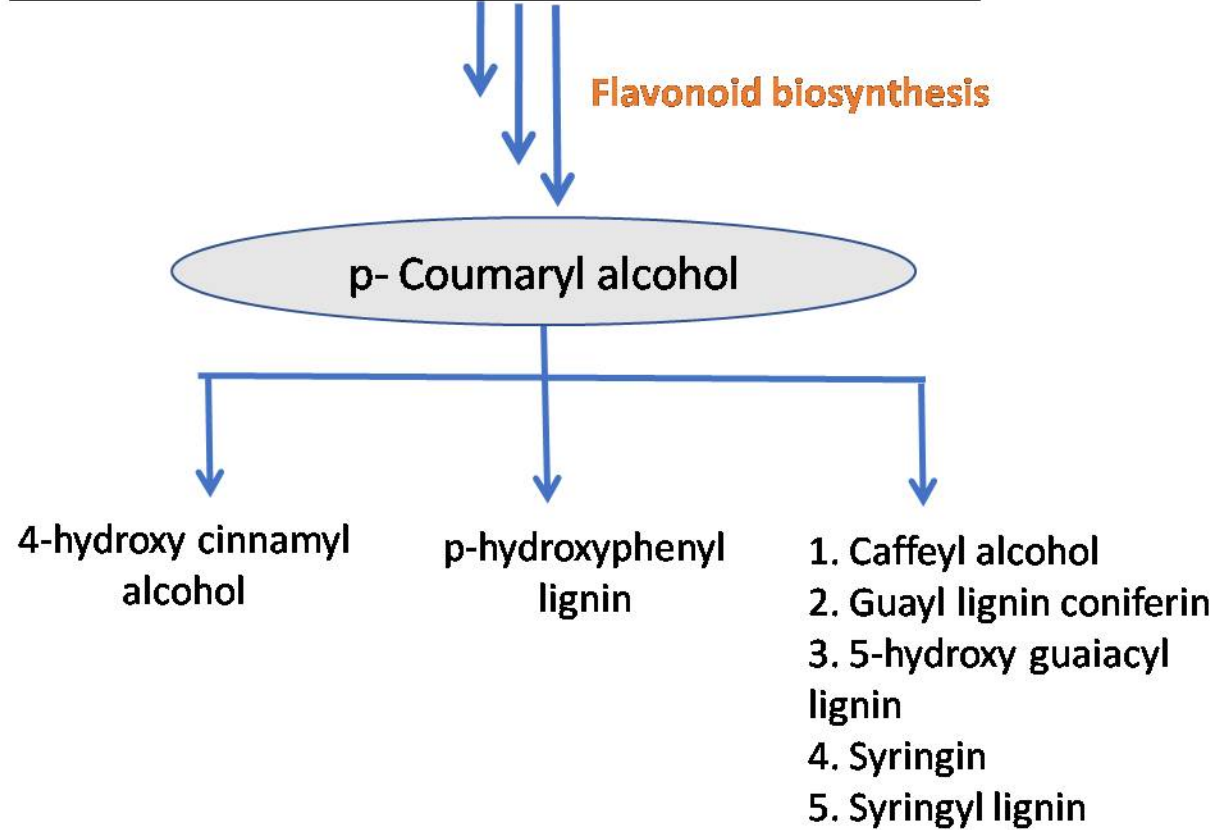**B**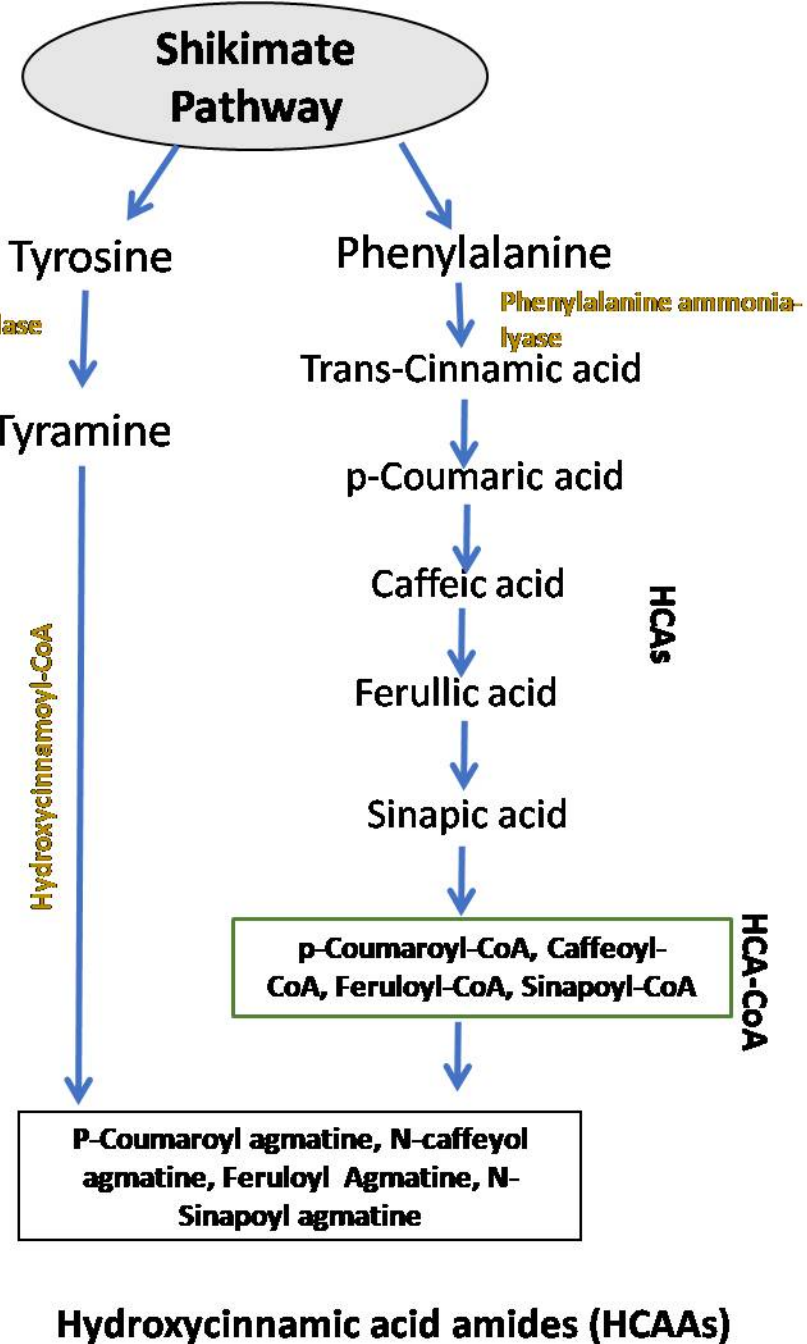

Supplement: Supplementary file 1 [file metabolites-16-00022-s001.zip › metabolites-3658309 Supplementary Fig 1.pdf]
